# Supplementary material for: Genetic Dissection of Drought Tolerance of Elite Bread Wheat (Triticum aestivum L.) Genotypes Using Genome Wide Association Study in Morocco
Source: Plants (Basel). 2022 Oct 13;11(20):2705. doi: 10.3390/plants11202705 (PMC9611990; doi:10.3390/plants11202705)
Supplement: Supplementary file 1 [file plants-11-02705-s001.zip › supplementary Figures.pdf]

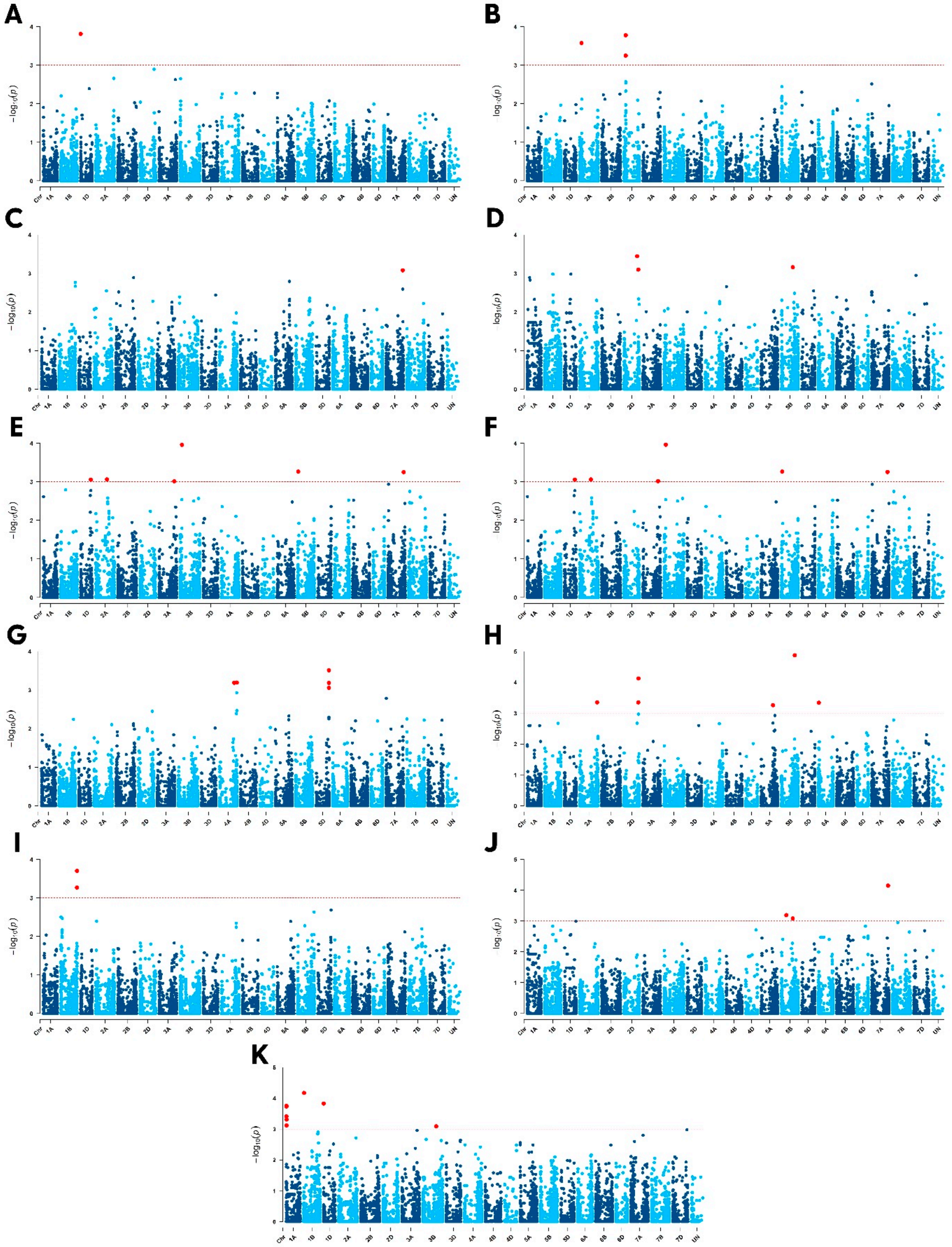

Figure S1: Manhattan plots of 200 bread wheat genotypes of DHE (A), DMA (B), PLH (C), GY (D), CT (E), CC (F), NSS (G), NPM (H), NTP (I), Biomass (J), and TKW (K) under irrigation conditions at Sidi Al-Aidi station.

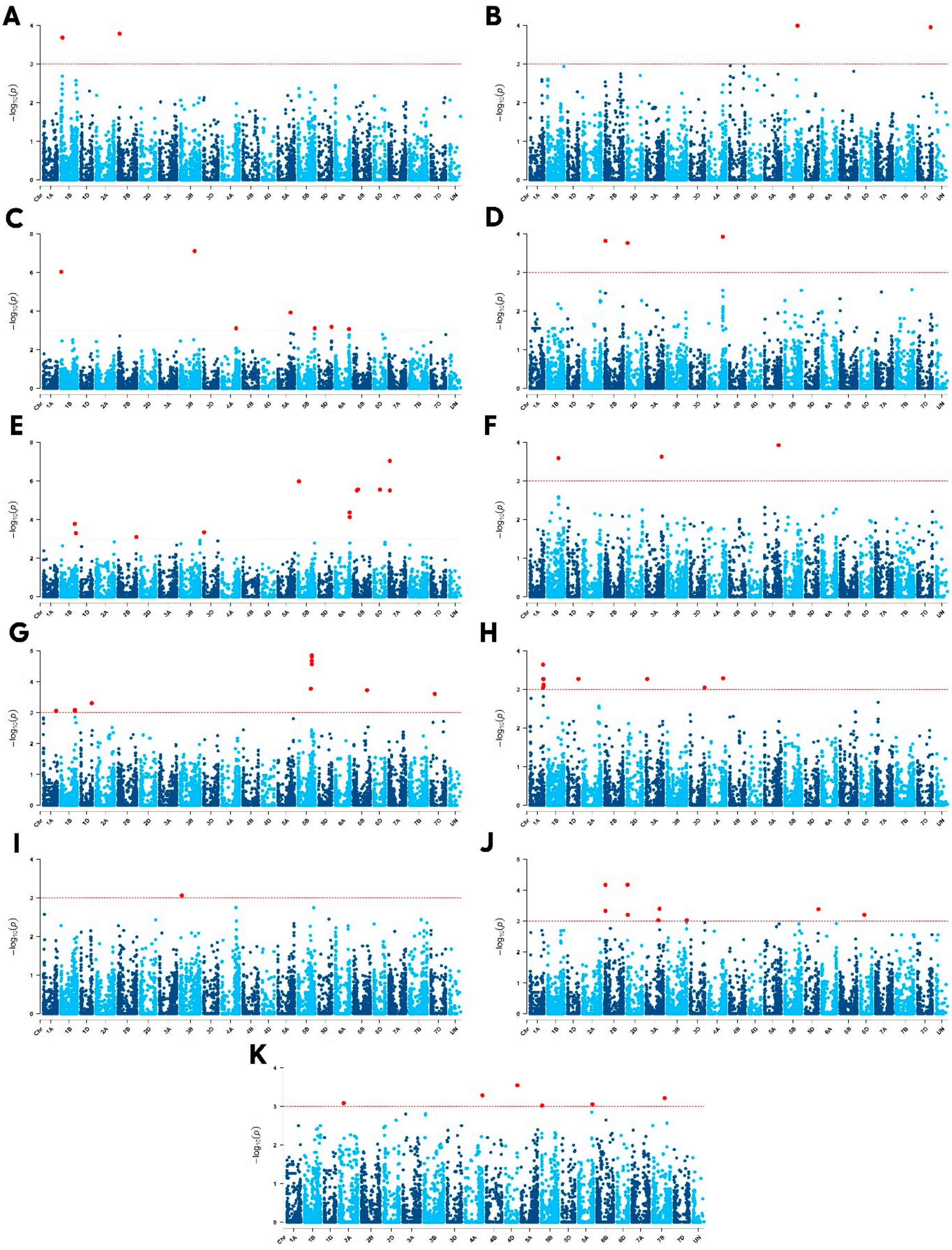

Figure S2: Manhattan plots of 200 bread wheat genotypes of DHE (A), DMA (B), PLH (C), GY (D), CT (E), CC (F), NSS (G), NPM (H), NTP (I), Biomass (J), and TKW (K) under rainfed conditions at Sidi Al-Aidi station.

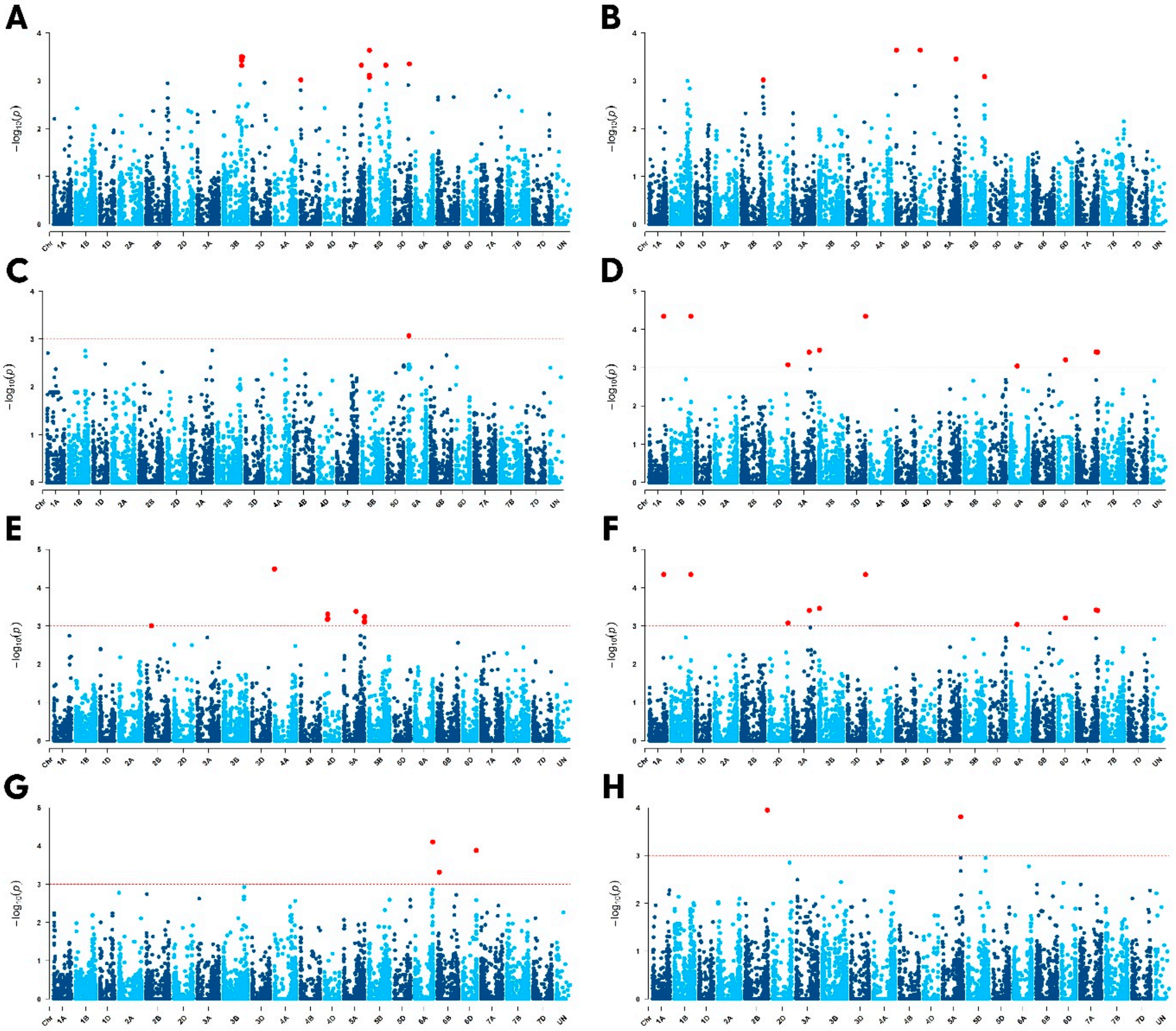

Figure S3: Manhattan plots of 200 bread wheat genotypes of DHE (A), DMA (B), GY (C), GY (D), CT (E), CC (F), Biomass (G), TKW (H), under rainfed conditions at Merchouch station.
